# Composition of receptor tyrosine kinase-mediated lipid micro-domains controlled by adaptor protein interaction

**DOI:** 10.1038/s41598-021-85578-8

**Published:** 2021-03-17

**Authors:** Arndt Rohwedder, Sabine Knipp, Lee D. Roberts, John E. Ladbury

**Affiliations:** 1 School of Molecular and Cellular Biology, University of Leeds, Leeds, LS2 9JT UK; 2 School of Applied Sciences, University of Huddersfield, Huddersfield, HD1 3DH UK; 3 Leeds Institute of Cardiovascular and Metabolic Medicine, University of Leeds, Leeds, LS29JT UK

**Keywords:** Membrane structure and assembly, Biochemistry, Biophysics, Cell biology, Molecular biology, Structural biology

## Abstract

Receptor tyrosine kinases (RTKs) are highly regulated, single pass transmembrane proteins, fundamental to cellular function and survival. Aberrancies in regulation lead to corruption of signal transduction and a range of pathological outcomes. Although control mechanisms associated with the receptors and their ligands are well understood, little is known with respect to the impact of lipid/lipid and lipid/protein interactions in the proximal plasma membrane environment. Given that the transmembrane regions of RTKs change in response to extracellular ligand binding, the lipid interactions have important consequences in influencing signal transduction. Fibroblast growth factor receptor 2 (FGFR2) is a highly regulated RTK, including under basal conditions. Binding of the adaptor protein, growth factor receptor-bound protein 2 (GRB2) to FGFR2 prevents full activation and recruitment of downstream signalling effector proteins in the absence of extracellular stimulation. Here we demonstrate that the FGFR2-GRB2 complex is sustained in a defined lipid environment. Dissociation of GRB2 from this complex due to ligand binding, or reduced GRB2 expression, facilitates the dispersion of FGFR2 into detergent-resistant membrane (DRM) micro-domains. This modification of the plasma membrane proximal to FGFR2 provides a further regulatory checkpoint which controls receptor degradation, recycling and recruitment of intracellular signalling proteins.

Diffusion through, and localisation within the plasma membrane is important for the control and regulation of receptor tyrosine kinase (RTK) signal output in both the absence and presence of extracellular stimulation^1–5^. The plasma membrane is a highly complex and dynamic environment. The heterogeneous outer and inner leaflet surfaces of the membrane are composed of numerous distinct domains, including separated phases of lipids and accompanying proteins which, at physiological temperatures, are often unstable and transient. The literature on partition of signalling molecules into defined regions of the plasma membrane lacks broad consistency largely due to the complexity of the components, the potential for inner and outer leaflets to sustain different phases, and the large number and variety of modes of signalling proteins supported by the plasma membrane^1^.

Direct visualization of protein-lipid micro-domains in model bilayer membranes has provided a tangible proof for the coexistence of liquid-ordered and liquid-disordered phases^2–6^. Many signalling molecules have been shown to be enriched within detergent-resistant membrane (DRM) fractions^2,3^. These micro-domains are characterised by their lipid molecular content, including cholesterol and sphingolipids^7^. The inclusion of the membrane protein flotillin has also been endorsed as a marker for DRM micro-domains^8,9^. DRMs typically range from 10 to 200 nm in diameter and have lifetimes typically in the region of µs-ms, but can also prevail up to minutes. The concentration of specific lipids within these domains results in distinct properties associated with higher molecular packing, extended lipid acyl chains, and reduced diffusivity^1,9^.

Many RTKs become associated with lipid micro-domains/DRMs and are organised in clusters^10–12^. These micro-domains provide a common platform within which ‘membrane single pass’ RTKs have been found to accumulate and interact with other proteins associated with downstream signal transduction^13,14^. Association of transmembrane (TM) proteins to lipid micro-domains reduces their ability for individual lateral membrane diffusion, thus providing temporal control of localization and an extended opportunity for productive weak protein–protein interactions^15^.

Fibroblast growth factor receptor 2 (FGFR2) is a particularly good example of a highly regulated RTK. In the absence of extracellular growth factor stimulation, FGFR2 exists as a preformed dimer that is stabilised by the presence of growth factor receptor-bound protein 2 (GRB2)^16^. GRB2, which is a constitutive dimer in its unphosphorylated form^17^, consists largely of two Src homology 3 (SH3) domains which sequentially sandwich an SH2 domain. Each GRB2 molecule of the dimer binds to a proline-rich motif in the C-terminal region of the receptor via its C-terminal SH3 domain. The presence of GRB2 in the resulting heterotetramer renders the receptor signalling-incompetent. Depletion of GRB2 in non-stimulated cells leaves the proline-rich motif of the receptor exposed to competition for binding from other SH3 domain-containing proteins. For example, phospholipase Cγ1 (PLCγ1) is recruited and becomes activated on binding to the receptor via its SH3 domain^18^. This activation in the absence of extracellular RTK stimulation leads to initiation of signals for increased cell motility and metastatic outcome, as well as up-regulation of the AKT signalling pathway resulting in cell proliferation and tumour growth^19,20^. On stimulation of the receptor, the kinase activity of FGFR2 is up-regulated^21^. The resulting phosphorylation of GRB2 results in dimer dissociation, and subsequent release from the receptor. GRB2 is unable to bind directly to the activated receptor^17^. With extracellular growth factor bound, and in the absence of the inhibitory adaptor protein, the fully active dimeric receptor trans-autophosphorylates tyrosine residues providing recruitment sites for downstream effector proteins.

The membrane localisation of FGFR2 and the impact of the TM region on dimerization in both the absence of growth factor, and on stimulation have been reported^22,23^. The importance of receptor-mediated control of membrane environment is further underscored based on the selectivity of signalling-associated proteins to lipid micro-domains^24^. For example, activation-dependent localization of known effectors of FGFR2, such as H-Ras, R-Ras and PI3K^25,26^, to lipid micro-domains has emphasized the importance of these signalling platforms and their constituents. However, there is limited understanding of micro-domain composition and receptor distribution in the membrane, or how this is affected by the presence of GRB2.

Here we show that the micro-domain clustering and subcellular distribution of FGFR2 depends strongly on GRB2 in the absence and presence of stimulation. This renders the adaptor protein/RTK binding an excellent model for the analysis of micro-domain formation, and the process ultimately leading to the assembly of signalling clusters. In the absence of growth factor, GRB2 binding regulates FGFR2 spacing within preformed membrane clusters which are diverse in phospholipid constituents. Removal of GRB2 from FGFR2, through extracellular stimulation or depletion of expression, results in FGFR2 appearing in DRMs with distinct lipid components from which downstream signalling can be initiated. Thus, the local lipid environment responds to the presence of the adaptor protein. Regulatory mechanisms associated with the control of entry to the appropriate membrane micro-domain are likely to be a common additional feature in control of other RTK-mediated signalling.

## Results

### The phospholipid composition of FGFR2-associated clusters is affected by the presence of GRB2

**Figure 1:**
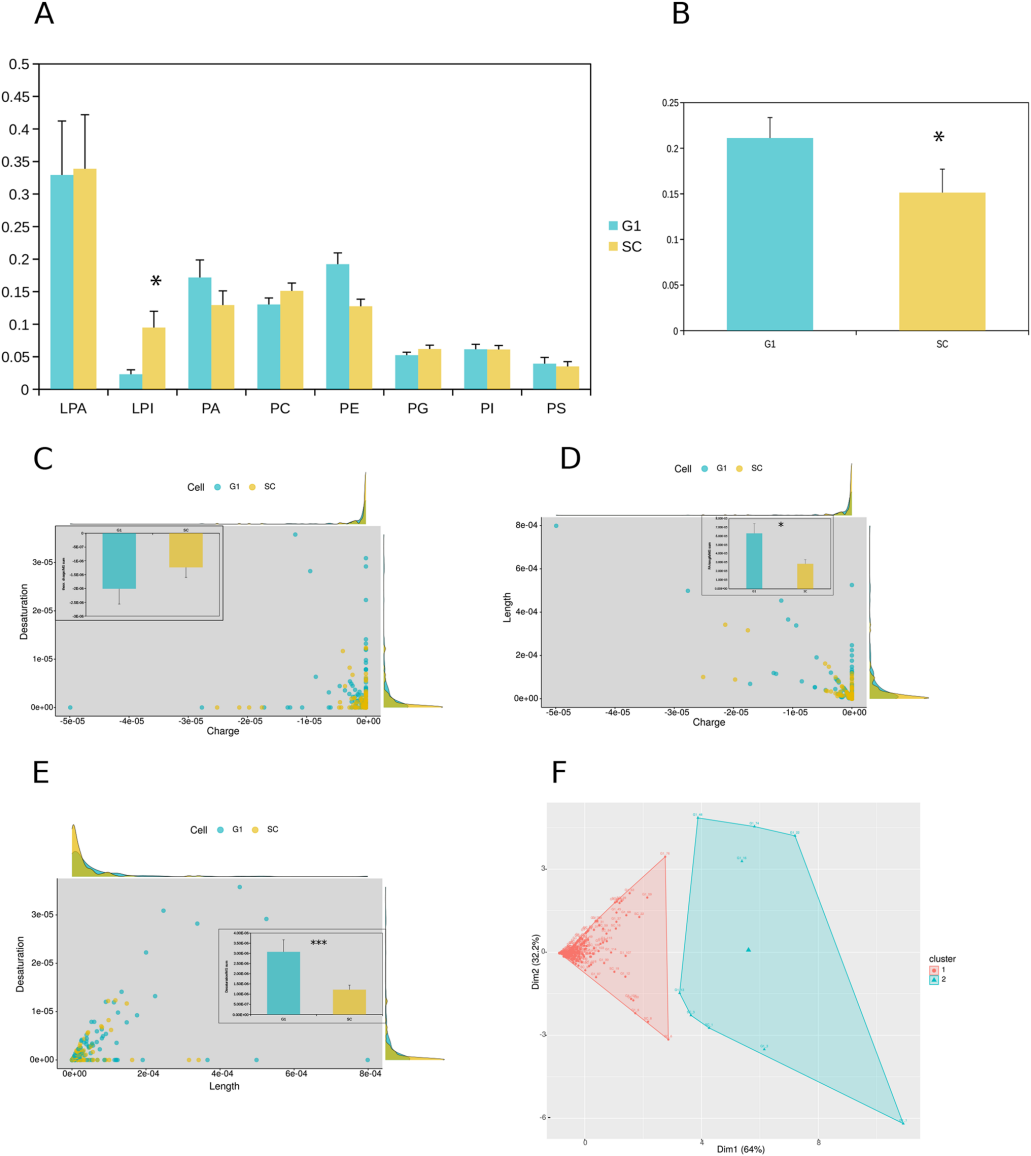
Lipid distribution in FGFR2-associated micro-domains. (**A**) Comparison between GRB2-depleted (G1) and control (SC) of identified lipid classes from mass spectrometric measurements of FGFR2 DRM extracts. The data has been normalized to the summarized signal of each of 3 replicates (see Materials and Methods). Error bars represent standard deviation. (**B**) Relative cholesterol content of DRMs from G1 and SC. (**C**) Scatter blot with densitogram of calculated signal adjusted charge (x-axis) versus fatty acid desaturation (y-axis) of identified phospholipids between G1 and SC. Insert: bar graph of the average adjusted head group charge. (**D**) Scatter blot with densitogram of calculated signal adjusted charge (x-axis) versus signal adjusted chain length (y-axis). Insert: bar graph of the average fatty acid chain length, *p* = 0.019. (**E**) Scatter blot with densitogram of calculated signal adjusted fatty acid chain length (x-axis) versus fatty acid desaturation (y-axis) of identified phospholipids between G1 and SC. Insert: bar graph of the average fatty acid desaturation, *p* = 0.000516. (**F**) Scatter blot representation of dimension 1 and 2 from k-means cluster analysis using ward method based on charge, fatty acid length and saturation. Cluster 2 consists predominantly of G1 data.

To investigate whether the presence of GRB2 has an impact on plasma membrane formation proximal to FGFR2, we used CRISPR/cas9 to knock out GRB2 in HEK293T cells transfected with FGFR2. We were able to achieve efficient GRB2 depletion (G1) compared to control (SC) cells (Supplementary Fig. S1A). The lipid composition from FGFR2-trapped membrane fractions derived from micro-domain isolates from these cells was measured by mass spectrometry (MS). We were able to identify nine major lipid classes: lysophosphatidic acid, LPA; lysophosphatidylinositol, LPI; phosphatidic acid, PA; phosphatidylcholine, PC; phosphatidylethanolamine, PE; phosphatidylglycerol, PG; phosphatidylinositol, PI; phosphatidylserine, PS and cholesterol. The composition of some of the lipid classes pulled down with the receptor were altered in the presence or absence of GRB2 (Fig. 1A). Under basal conditions in the presence of the FGFR2/GRB2 complex, LPI was significantly more frequently found to be associated with the receptor. On removing GRB2 the distribution of major phospholipid membrane components around FGFR2 changed, with a relative increase in cholesterol species being significant (Fig. 1B). 2D-HPTLC of the micro-domain environment from membrane fractions including FGFR2 confirmed the relative reduction of PC compared to cholesterol in GRB2-depleted cells (Supplementary Fig. S1B). MS allowed the identification of phospholipid species (Supplementary Fig. S2) and statistical analysis of the fatty acid chain composition (Fig. 1C-F). We supplemented these data using the LipidMaps database (https://www.lipidmaps.org/) to assign the nominal charge for the phospholipid head-groups. These changes correlate with a shift toward an accumulation of negative charges in the GRB2-depleted cells (Fig. 1C,D). Chain length and desaturation of the phospholipid fatty acid chains were significantly higher in the GRB2-depleted cells compared to control cells (inserts Fig. 1D,E). Applying k-means cluster analysis to the combined data from chain length, desaturation and charge, revealed separation into two clusters, with cluster 2 being dominated by data from GRB2-depleted cells (Fig. 1F). This shows that the lipid environment proximal to FGFR2 in the plasma membrane differs significantly in the presence or absence of GRB2.

### GRB2 modulates clustering and juxtaposition of FGFR2 in micro-domains

**Figure 2:**
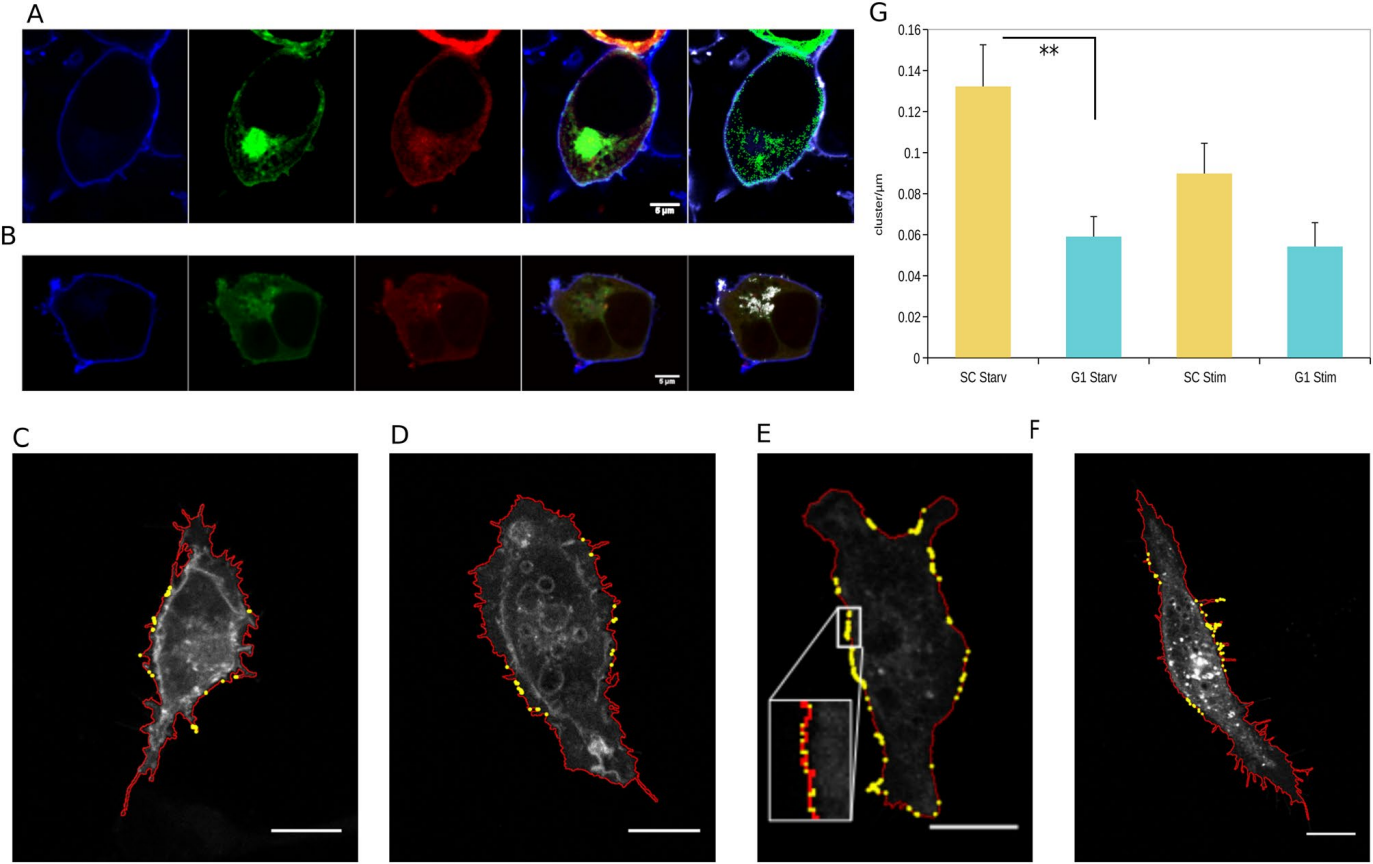
Microscopic characterisation of FGFR2 clusters under starvation and on stimulation in the presence and absence of GRB2. (**A**–**B**) Distribution of FGFR2 and GRB2 at the plasma membrane. (**A**) HEK293T serum starved, (**B**) HEK293T FGF9 stimulated. Left to right: membrane stained with DiB, FGFR2-GFP, GRB2-mRFP, overlay, co-localisation (clusters: white dots). Scale bar = 5 µm. (**C**–**F**) Confocal microscopic images of single z-slice of plasma membrane localized FGFR2-mCherry and cholesterol-Cy5 cluster based on the ImageJ Cluster plugin; membrane (red) and size superimposed clusters (yellow). (**C**) Control (SC) serum-starved. (**D**) GRB2-depleted (G1) serum starvation. (**E**) Control (SC) 20 min. after FGF9 stimulation. Inset shows expanded image of spacing of clusters demonstrating that although there appears to be a large number of clusters in the image they are distal from one another. (**F**) GRB2 reduced (G1) 20 min. after FGF9 stimulation. Scale bar = 5 µm. (**G**) Quantification of identified cluster/µm in SC and G1 cells under serum starvation and in SC and G1 20 min. after FGF9 stimulation. Significant difference between SC and G1 starvation (*p* = 0.004).

Having demonstrated that the presence of GRB2 has a profound impact on the components of the micro-environment around the receptor, we investigated the relationship between FGFR2 and GRB2 proximal to the plasma membrane. HEK293T cells were doubly transfected with C-terminally GFP-tagged FGFR2 (FGFR2-GFP) and C-terminally RFP-tagged GRB2 (GRB2-RFP) and subsequently stained with DiB to highlight the plasma membrane. In the absence of growth factor, both FGFR2 and GRB2 distribute at the membrane with both proteins co-localizing into discernible clusters (Fig. 2A). It is of interest to note that the cells also display an intense region of FGFR2-GFP proximal to the nucleus. This appears to be due to accumulation of the receptor in the Golgi. On stimulation we saw a reduction in overall concentration of co-localised FGFR2 and GRB2 (Fig. 2B), consistent with the dissociation of the heterotetramer on growth factor binding^16^. In addition, there was evidence that both FGFR2 and GRB2 were internalised and co-localised in a region that is proximal to the nucleus, in agreement with previous observations of stimulation-dependent endocytosis of FGFR2^17^.

To assess whether GRB2 influences the clustering of FGFR2, we used an unbiased method for identifying the presence of discrete clusters of FGFR2 on the plasma membrane. For this we developed a plugin tool for ImageJ that detected the plasma membrane and quantified the fluorescently labelled protein-containing clusters localised within (modified from a cell surface morphology tool^27^ (Cluster 19 plugin. Supplementary Fig. S3)). By scanning the circumference of parallel slices of the cell and retaining the positions of detected signals within a selected threshold, clustering of proteins was quantified. This produced a picture of cluster intensity across the entire cell surface. A representation of the detected plasma membrane is generated as a control in a separate image. The output from the tool provides a measure of the number of clusters/µm of cell circumference for a pre-determined number of cell slices.

We had previously observed the presence of FGFR2 in a cholesterol-enriched membrane environment in both the absence and presence of GRB2 (Fig. 1B). To observe the appearance of clusters of the receptor, HEK293T cells were transfected FGFR2-mCherry such that we could observe co-localisation in clusters with Cy5-labelled cholesterol. The images of single slices of representative cells reveal the impact of the presence of GRB2 in both serum-starved and FGF9-stimulated cells (Fig. 2C-F and quantified graphically in Fig. 2G). Under serum-starved conditions FGFR2 clustering in the presence of cholesterol was present on the plasma membrane. This receptor clustering was significantly reduced in GRB2 knockdown (G1), compared to the control (SC) cells (Fig. 2G), suggesting that GRB2 influences the self-association of FGFR2 molecules. Under stimulated conditions the absence of GRB2, due to phosphorylation by the receptor (SC cells) or deprivation of expression (G1 cells), resulted in a dramatic reduction in the amount of identified clusters (Fig. 2G). It is important to note that the observed receptor clustering does not correlate with downstream signalling capability, i.e. clustering per se is not a marker of signalling competency.

**Figure 3:**
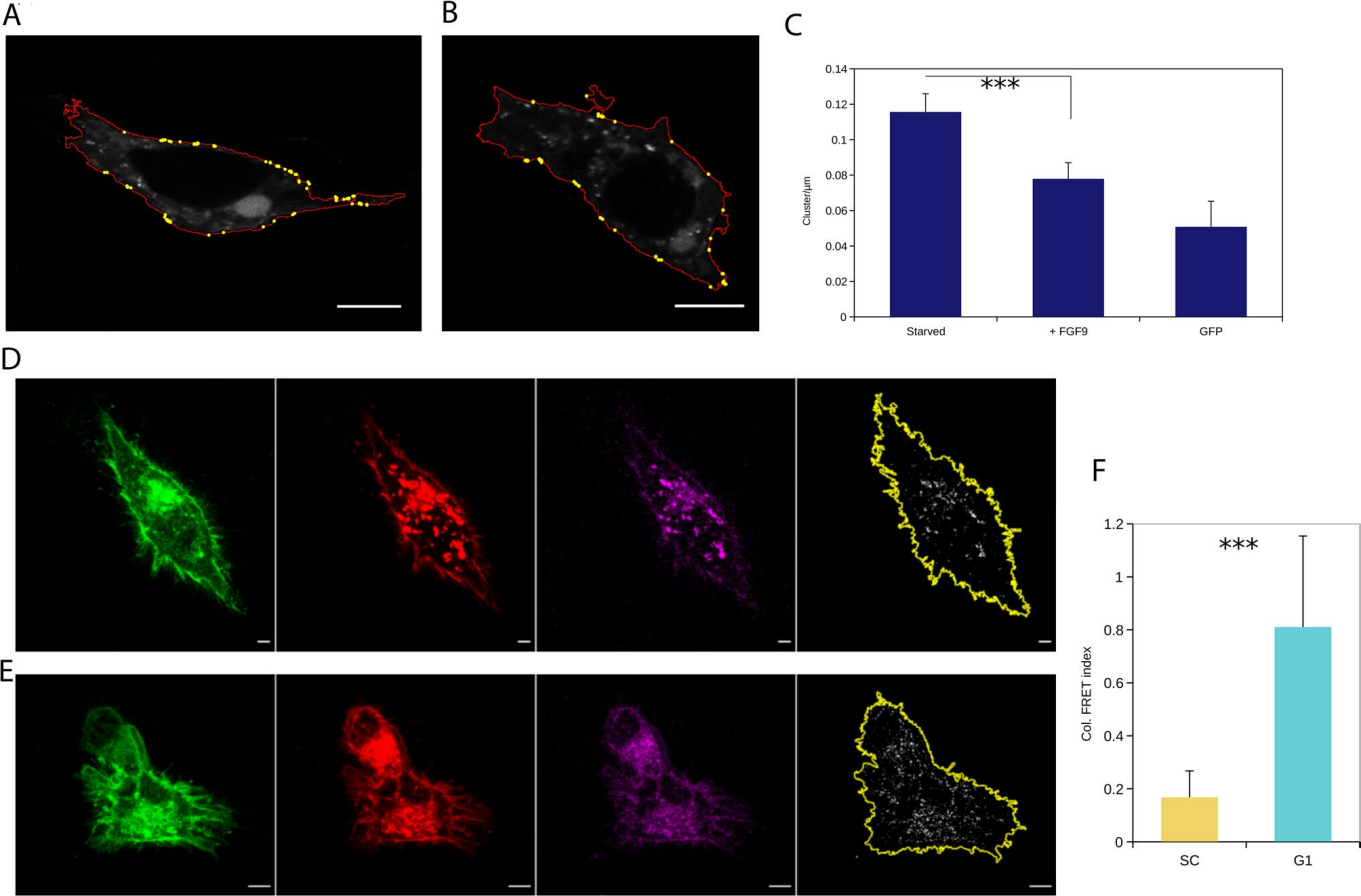
(**A**–**C**) Cluster identification of GRB2-split GFP dimer at the plasma membrane. (**A**) Serum starved, (**B**) 20 min. after FGF9 stimulation. Scale bar = 5 µm. (**C**) Quantification of GRB2 dimer clusters per µm. Significant difference between serum starvation and FGF9 stimulation (*p* = 0.0006). (**D**–**F**) FRET measurement of serum starved HEK293T transfected with FGFR2-GFP and FGFR2-mCherry to determine dimerisation of receptor. (**D**) Control cells (SC), (**E**) GRB2-depleted cells (G1). Left to right: Donor (FGFR2-GFP), Acceptor (FGFR2-mCherry), FRET signal, integrated FRET area. Scale bar = 5 µm. (**F**) Quantification of integrated complete FRET area (*p* <  = 0.001).

The impact of GRB2 on FGFR2 self-association is affected by its ability to dimerise. We were able to confirm that GRB2 was in the form of a dimer at the plasma membrane using fluorescence signal derived from reconstitution of a split GFP fusion tag on GRB2. Quantification with the Cluster 19 plugin confirmed the presence of clustered populations of GRB2 dimers on the membrane under serum-starved conditions (Fig. 3A), consistent with the adaptor protein being in a heterotetramer with FGFR2^16^. On stimulation with FGF9 the number of clusters/µm on the membrane decreased, in line with the disassembly of the FGFR2-GRB2 complex on up-regulation of the receptor (Fig. 3B,C).

Fluorescence resonance energy transfer, FRET, data from serum-starved HEK293T cells transfected with both C-terminally labelled FGFR2-GFP and FGFR2-mCherry showed the impact of GRB2 on the juxtaposition of the FGFR2 molecules in the presence, and absence of the adaptor protein. We observed fluorescence emission from the membrane of SC cells in which FGFR2 and GRB2 can form the heterotetrameric complex (Fig. 3D). Depletion of the adaptor protein in G1 cells led to an increase in FRET emission (Fig. 3E,F). This indicated that, in the presence of GRB2, the distance between the fluorophores, and hence the spacing between the C-terminal ends of two FGFR2 monomers, is sufficient to prevent FRET (> 10 nm), whilst depletion of GRB2 results in closer self-association of the receptors^28^. Thus, although GRB2 is able to enhance FGFR2 clustering, the adaptor protein appears to hold the receptor molecules in a conformationally distanced state within the context of the heterotetramer.

### The presence of GRB2 prevents FGFR2 distribution in DRMs

**Figure 4:**
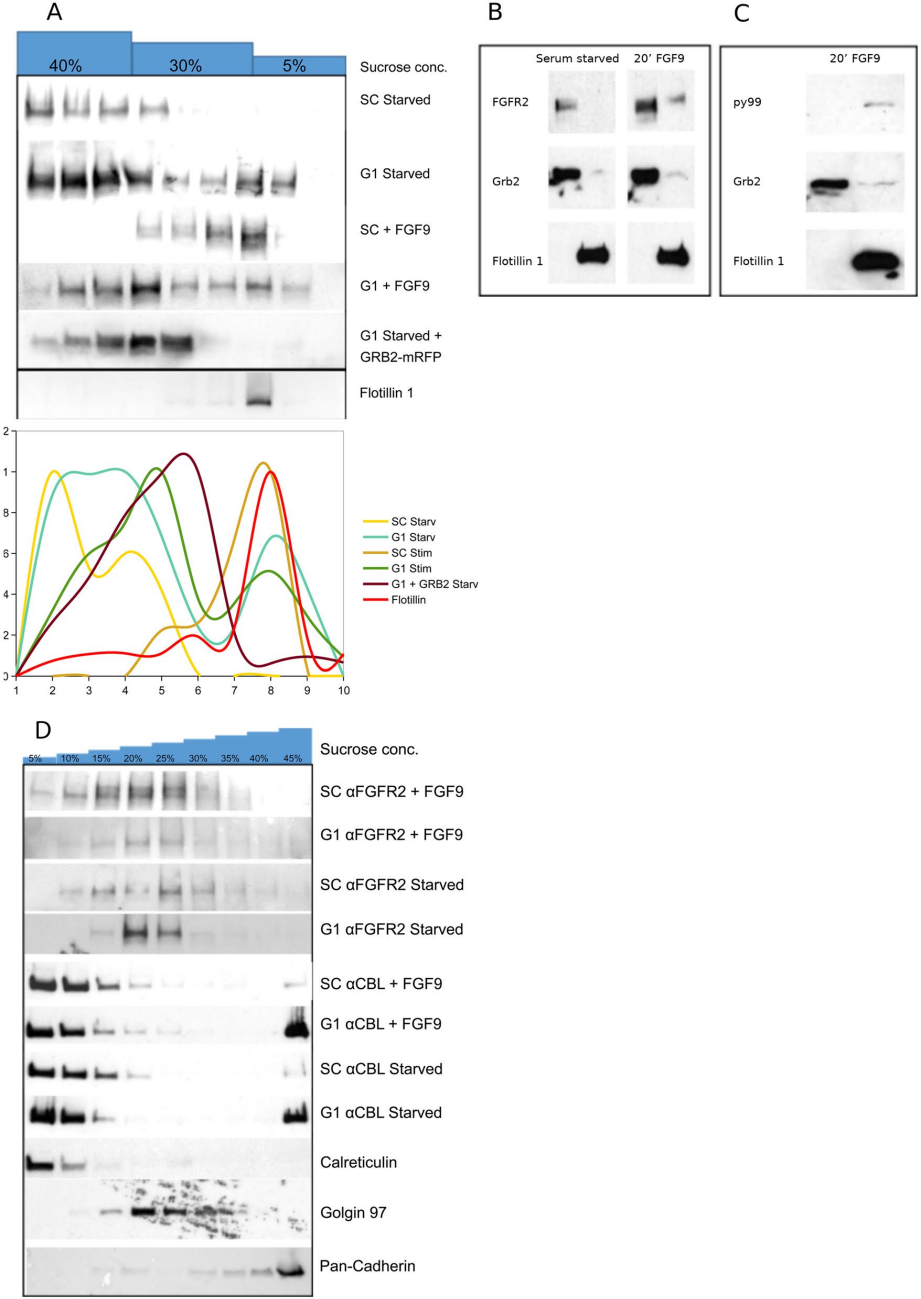
Biochemical DRM characterisation of FGFR2 distribution. (**A**) Sucrose gradient DRM separation showing the presence of FGFR2 in different sucrose concentrations. Flotillin-1 indicates DRM fraction. Separated panel shows distribution of Flotillin 1 across identical sucrose gradient. Line graph below shows normalized signal distribution along gradient. (**B**–**C**) Two step fractionation showing supernatant (first lane of individual blots) and pellet (second lane) fractions. Flotillin-1 marker DRM fraction (in pellet). (**D**) Sucrose gradient cell fractionation. An anti-CBL antibody was used to show fractional localisation of CBL in starved SC and G1 cells in the absence and presence of FGF9 stimulation. Calreticulin = ER marker, Golgin-97 = Golgi marker, pan-Cadherin = plasma membrane marker. Sucrose gradient blots are loaded on separate gels. The original blots are included in Supplementary Figure S5.

Our data suggest that the micro-environment experienced by FGFR2 in the presence and absence of GRB2 is different. Further evidence of the effect of GRB2 on the micro-environment mediated by FGFR2 in the plasma membrane was provided by using ultracentrifugation to separate detergent treated membrane fractions. Using sucrose gradients with flotillin as the marker, we found that the receptor was present in DRMs in serum-starved HEK293T cells transfected with FGFR2 in the absence of GRB2 (G1 cells, Fig. 4A), but not in the presence of GRB2 (SC cells). On stimulation, phosphorylated FGFR2 (as shown by the pY99 antibody, Fig. 4B and Supplementary Fig. S1C) also appeared in the DRM membrane fraction (Fig. 4A and Fig. 4C). Thus the absence of GRB2, whether associated with depletion or through FGFR2 up-regulation, coincides with the inclusion of FGFR2 in DRM micro-domains. This observation is consistent with the MS data that show, in the absence of GRB2, the micro-environment in which FGFR2 is localized contains lipids with lower net charge, extended lipid chains and hydrophobicity; all features of the DRM environment. The broad distribution of FGFR2 throughout the gradient from serum-starved cells in the absence of GRB2 is comparable to that seen on stimulation (Fig. 4A). The effect of distribution of FGFR2 in DRMs in G1 cells was reversed by the addition of GRB2 through transfecting cells with GRB2-mRFP (Fig. 4A).

### Depletion of GRB2 affects the intracellular transport and organelle association

To discern whether the depletion of GRB2 might influence the cellular localisation of FGFR2, discontinuous sucrose gradient-based microsome fractionation was adopted to characterise subcellular localization of FGFR2 in HEK293T cells expressing the receptor. Comparison of the distribution of FGFR2 across the gradient indicated that, in serum-starved cells the presence of GRB2 appears to increase the amount of FGFR2 retained in the membrane marked by pan-Cadherin (Fig. 4D). The broad distribution of FGFR2 across the gradient seen in Fig. 4A is also observed. The depletion of GRB2 led to the presence of FGFR2 in fractions containing the Golgi marker, Golgin 97. Faint bands are observed at the high sucrose concentrations which are absent in the presence of GRB2 consistent with plasma membrane localisation with pan-Cadherin. This confirmed previous observations on membrane localisation, but also suggested that, under basal conditions when GRB2 is depleted, the receptor is recycled via endocytosis and localisation to the Golgi. In other words, GRB2 limits receptor internalisation and processing.

GRB2 depletion in the absence of FGF9 led to substantial localisation of the ubiquitin ligase, CBL to the membrane fractions (marked by pan-Cadherin), suggesting that ubiquitinisation and subsequent degradation of FGFR2 might occur (Fig. 4D). The presence of CBL in the FGFR2 membrane-localised fractions with reduced GRB2 was also apparent in the FGF9-stimulated cells, consistent with GRB2 not only restraining the non-signalling receptor from DRM-microdomains, but also preventing ubiquitination and intracellular protein degradation.

## Discussion

Lipid micro-domains have been described as platforms that support RTKs and other TM proteins to form a "signalosome"^8^. Thus, through control of the appearance of RTKs in these micro-domains, regulation of signal transduction can be imposed. Our observation of significant changes in the lipid composition of membrane fractions accompanying FGFR2, in the presence and absence of GRB2, highlight a novel receptor regulatory mechanism.

MS of the lipid classes in fractions from cells under serum-starved conditions revealed differences in the membrane constituents (Fig. 1A). In the absence of GRB2 the micro-domains have increased levels of cholesterol (Fig. 1B), reduced net charge (Fig. 1C) and, most significantly, the receptor is in an environment with increased chain length and hydrophobicity (Fig. 1E). All of these features are consistent with the receptor, when not associated with GRB2, sustaining a DRM environment^1,9^.

On stimulation, a reduction in the number of receptor clusters was observed (Fig. 2C-G). The binding of FGF9 up-regulates the kinase activity of the receptor leading to dissociation of GRB2 from FGFR2^16^ (Fig. 3A-C). These conditions closely resemble those seen in cells with depleted expression levels of the adaptor protein where the receptor also sustains DRM fractions (Figs. 4A). Thus, removing the interaction between FGFR2 and GRB2 permits the receptor to interact with the lipids found in DRMs which exhibit higher molecular packing, extended acyl chains, and reduced diffusivity^1,9^.

Depletion of GRB2 in serum-starved cells results in the receptor within the DRM being able to interact with downstream signalling proteins. It is possible that the lipid environment has an impact on recruitment of other SH3 domain-containing proteins and hence, in the absence of GRB2 the receptor is released from a lipid environment that could be considered as a ‘silencing platform’. The presence within the DRM micro-environment is also fundamental to the recruitment of the ubiquitin ligase CBL to the plasma membrane by FGFR2^29^ (Fig. 4D). Thus, through regulation by GRB2, the membrane environment proximal to the receptor has a dramatic impact on signalling capability, and the processing of the receptor.

The absence of GRB2 appears to release FGFR2 from a state in which its C-terminal regions are spatially restrained from one another preventing FRET of attached fluorophores (Fig. 3A-C). This suggests that a structural perturbation of the receptor accompanies dissociation of the adaptor protein. Based on the different membrane environments experienced by the receptor, we propose that this conformational change also involves modification of the TM region of the receptor to permit association with phospholipids with extended chains found in the DRM. This is consistent with previously reported growth factor binding resulting in pronounced changes in the TM domain of FGFR2^23^. Further possible support for this proposal is apparent in the conservation of the sequences of TM regions of FGFR2 across species (Supplementary Fig. S4). This conservation of TM regions throughout evolution highlights the requirement of the receptor to sustain a DRM capable of a unique signalosome, and hence cellular function. This contrasts with the different TM sequences seen for different FGFR family members which require different DRM components for their signalling function.

We therefore, propose a model where the lipid environment of FGFR2 is influenced by the conformational state of the receptor itself. In the absence of stimulation, the FGFR2 dimer remains in complex with GRB2 and adopts an inactive conformation in the plasma membrane. In this conformation the TM region is unable to bind to the elongated DRM-associated lipids. Upon stimulation, and concomitant dissociation of GRB2, the receptor TM in the context of the ligand bound dimer undergoes conformational change allowing association with a different lipid composition of the micro-environment around the TM region. The more ordered lipid environment of the DRM has an impact on the receptor’s ability to recruit downstream effector molecules and initiate kinase activity. An example of this is seen with FGFR3 where localisation of the receptor in membrane regions of increased thickness decreases the tilt angle of the TM helix, bringing it closer to the membrane normal resulting in the release of the juxtamembrane (JM) region from the membrane, and therefore promoting activation of the receptor^30^. In addition, the observed change in electrostatic conditions of the components of the DRMs observed potentially provide an anionic membrane inner surface which would be repellent to a progressively phosphorylated kinase domain, enhancing the dynamic, asymmetric dimerization required for trans-autophosphorylation. When GRB2 is depleted from the cell, the TM is constitutively able to bind to the lipid constituents with elevated negative charge and extended chains such that the lipid environment is similar to the post-stimulation DRM. It appears therefore, that GRB2 prevents the receptor from undergoing the conformational change required to be associated a DRM. Without GRB2 the receptor is in a signalling-competent lipid environment, however, the lack of tyrosyl phosphate sites prevents canonical RTK signal transduction. In this case the absence of GRB2 allows recruitment of proteins to the exposed proline-rich site (e.g. PLCγ)^19,20^.

The observed association of FGFR2 with DRMs enables up-regulation and access to a downstream effector protein-enriched platform^25,26^. Thus, the presence of GRB2 bound to the C-terminal region of FGFR2, not only physically blocks recruitment of signalling proteins^18^, but adds a further level of inhibitory regulation through abrogating inclusion within DRMs. It is highly likely that lipid micro-domain clustering of TM proteins is a common feature of regulation, where the composition of the micro-domains change with the binding state of the protein. As a consequence this offers the opportunity to control signalling events by addressing the lipid micro-domains composition without effecting RTKs directly.

## Materials and methods

### Chemicals

Golgin 97 and Pan-Cadherin from Cell Signalling Technology. Calreticulin from Genetex. Py99, FGFR2, Flotillin-1, GRB2 antibodies from Santa Cruz. c-CBL from ThermoScientific. Lipid standards were from Larodan, Sweden. Solutes for HPTLC were from Sigma.

### Cell culture and protein knock down

HEK293T cells were grown with DMEM (Gibco) with 10% FBS and 1% penicilin/streptomycin. For serum starvation cells were washed with PBS and grown overnight with DMEM with 1% penicillin/streptomycin. For stimulation 20 ng/ml FGF2/FGF9 was used. To ‘knock down’ GRB2 in HEK293T cells CRISPR (Blue Heron BioTech) was used and positive cells were selected with puromycin (Life Technologies). cCBL was transiently silenced with CBL-SiRNA (Santa Cruz). Split GRB2-GFP was generated from GRB2-mCherry and pEGFP using Q5 mutagenesis (NEB). FGFR2c-mCherry was generated from Addgene FGFR2c (#45699).

### Confocal imaging and analysis

Membrane (DiB CellBrite Blue from Biotum) and cholesterol (Cholesterol-PEG-Cy5 from Ruixibio, 3-hexanoyl-NBD Cholestrol from Cayman Chemicals) stains were respectively applied to cells 5 min. prior to PFA fixation. Fixed cells were recorded using LSM700 with Plan-Apochromat 63x/1.40 Oil DIC M27 objective and instrument settings of the Zeiss ZEN software for Alexa Fluor 405, EGFP, and mCherry. The resulting images were analysed using the group-generated Cluster 19 plugin for imageJ (see Supplementary Fig. S3 for details). FRET measurements were performed as previously described^31^. Three images of the same cell and slice were acquired on the confocal microscope using identical magnification conditions, with the wavelength combinations GFP 488 nm/518 nm and mCherry 555 nm/585 nm, FRET 488 nm/585 nm.

### DRM isolation and separation

Cells were harvested in 1% Tween-20, 10 mM MgCl_2_ with protease inhibitors and homogenized with 20 strokes 0.8 mm needle followed by 15 strokes with 0.4 mm needle. After lysis for 2 h at 7 °C while shaking debris was removed by pelleting at 2000 × G for 15 min. at 4 °C. DRMs were than either pelleted for 90 min. at 100,000 × G or applied to a step gradient by mixing with 80% sucrose 1/1 followed by 30% sucrose and 5% sucrose on top. After ultracentrifugation at 100,000 × G, 4 °C for 90 min. the gradient was harvested in 500 µl steps. Proteins have been precipitated using 9 vol. methanol over night at 7 °C.

### Cell fractionation

Cells were harvested in 250 µM sucrose solution with protease inhibitors and subsequently homogenised using 20 glass piston strokes with homogenizer, 20 strokes with 0.8 mm needle, followed by 15 strokes with 0.4 mm needle. Debris was pelleted with 15 min 2000 × G at 4 °C. Resulting supernatant has been centrifuged at 10,000 × G for 30 min. at 4 °C to remove mitochondria. Further 90 min. centrifugation at 100,000 × G at 4 °C pelleted the microsomal fraction which had been homogenised with 0.25 mM sucrose and transferred on top of a step gradient consisting of 9 steps ranging from 45 to 5% sucrose. Separation was done by ultracentrifugation at 100,000 × G for 90 min or overnight at 4 °C.

### Lipid extraction and HPTLC

Lipids were extracted with modified Folch extraction: samples were homogenised in 170 µl PBS, 750 µl MeOH/chloroform/1 M HCl were added, vortexed, incubated at 25 °C for 5 min. additional 170 µl 1 M HCl and 750 µl chloroform was added. Phase separation has been facilitated with 5 min. centrifugation at 1000 × G at RT. The lower phase has been transferred, and washed using 708 µl MeOH/chloroform saturated 0.1 M HCl. Phase separation was enhanced by 5 min. centrifugation at 1000 × G at RT. The resulting lower phase was stored at -80 °C. Before use samples have been reduced to ~ 10 µl using N_2_ gas flow.

HPTLC was performed on HPTLC silica plates (LiChorsphere Silicagel 60) as described previously^32^. In short, plates were dried at 100 °C for 20 min., then washed with MeOH/ethylacetate (6/4), and heated to 110 °C for 30 min. Samples were applied 1 cm above lower edge of the plate and placed into a vapour saturated TLC chamber with TLC solution 1 (dichlormethan/ethylacetate/Acetone: 80/16/4). Upon reaching 10 cm plates were removed and air dried, then returned to the saturated TLC chamber with TLC solution 2 (chloroform/ethylactetate/acetone/isopropanol/ethanol/methanol/H_2_O/acetic acid: 30/6/6/6/18/28/6/2). After air-drying plates were sprayed with a 10% CuSO4/8% phosphatidic acid stain and developed for 10 min. at 145 °C. The resulting fluorescent stain was recorded on a G-box system using TAMRA setting.

### Lipidomics

N_2_ dried lipid samples were applied to mass-spectrometer and analysed. Lipidomic analysis was performed as previously described^33^. Briefly, samples were reconstituted in 100 μl liquid chromatography-mass spectrometry (LC–MS) grade pre-cooled isopropanol (4 °C) (Chromasolv, Fisher Scientific) and vortex-mixed and sonicated for 30 min. before being transferred to LC vials.

Chromatography was performed using an ACQUITY UPLC system (Waters Corporation) equipped with an ACQUITY UPLC BEH Amide 1.7 μm (2.1 × 100 mm) column, which was kept at 45 °C. The ACQUITY UPLC system was coupled to a Xevo TQ-XS mass spectrometer (Waters Corporation). The binary solvent system used was Solvent A comprising LC–MS grade acetonitrile:water (95:5), 10 mM ammonium acetate (Sigma-Aldrich) and Solvent B comprising LC–MS grade acetonitrile:water (50:50) (Chromosolv, Fisher Scientific), 10 mM ammonium acetate (Sigma-Aldrich). A 1 μl injection was used and mobile phase was set at a flow rate of 0.6 ml/min.

The column mobile phase was increased from 0.1 to 20% solvent B for 2 min followed by an increase from 20 to 80% solvent B over 3 min. The mobile phase was then re-equilibrated for 3 min. The total runtime was 8 min.

Analyses were performed using multiple reaction monitoring (MRM). The method analysed 493 lipid species (252 in positive mode, 241 in negative mode). Transitions and ionisation conditions are as previously reported^33^. The Xevo TQ-XS was operated in positive–negative switching electro-spray ionisation (ESI) mode. The positive mode capillary voltage was 2.8 kV and negative mode capillary voltage was 1.9 kV. The source temperature was 120 °C. A cone gas flow rate of 150 ml/h and desolvation temperature of 500 °C was used.

### Lipidomics data analysis

Data were processed and peak integration performed using the Waters Targetlynx application (Waters Corporation). The technical intra-individual sample CV for all species reported was equal to or less than 20%.

### Data analysis

Mass spectrometric chromatogram peak integrated data was normalized to the summarized signal (normalized to total lipid content) of each of 3 replicates. Individual lipid species were grouped into lipid classes and the total signal for each class summed. Statistical analysis of lipid class was performed using multiple *t* tests and corrected for multiple comparisons using the Holm-Sidak method. Data from the lipid mass spectrometric measurements were separated for chain length and saturation using R using the mclust package after normalization for relative appearance. Densitometry and scatter blot were generated using the ggpubr package. For lipid head group charge analysis the charge was derived from LipidMaps database (https://www.lipidmaps.org/). RStudio was used for Wilcoxon (Mann–Whitney) significance test.

## Supplementary information

Supplementary information.
