# Supplementary material for: Composition of receptor tyrosine kinase-mediated lipid micro-domains controlled by adaptor protein interaction
Source: Sci Rep. 2021 Mar 17;11:6160. doi: 10.1038/s41598-021-85578-8 (PMC7969938; doi:10.1038/s41598-021-85578-8)
Supplement: Supplementary file 1 — Supplementary information. [file 41598_2021_85578_MOESM1_ESM.pdf]

Composition of receptor tyrosine kinase-mediated lipid micro-domains controlled by adaptor protein interaction.

Arndt Rohwedder, Sabine Knipp, Lee D. Roberts, John E. Ladbury

Supplementary Figure Legends

Figure S1. A. Western blot of HEK293T cell lysates showing CRISPR/cas9 knockdown of GRB2. Blot probed with anti-GRB2 antibody. Viniculin shown as loading control. SC = matched control HEK293T cells with scrambled GRB2 RNA. G1 = HEK293T cells with GRNA1 against Grb2. WT = wild type cells without any genetic modification. **B**. Independent quantification of PC. Iodine vapour stained 2D HPTLC of lipid extracts from serum starved SC (upper image) and G1 (lower image) HEK293T cells. Staining intensities highlighted/enhanced using rainbow LUT in imageJ. Standard-identified PC marked with magenta circle. **C.** Blot showing that PY99 antibody interacts with pY site on FGFR2.

Figure S2. Relative distribution of identified species of phospholipids in FGFR2-specific DRMs from HEK293T SC and G1 cells.

Figure S3. Detailed workflow for Cluster 19 ImageJ Plugin. Open Graphic User Interface (GUI): Allows the user to change measurement relevant parameters: 1) Threshold for grey levels; 2) Confidence interval for co-localization; 3) Channel 1 to analyse; 4) Channel 2 to analyse.

The plugin then generates a working copy of stack and initializes a region of interest (ROI) using whole image. For detection the channels are combined to one grey image

internally. Filters are applied to this: auto-threshold and noise removal. Next the plugin calls the Particle Analyser function to identify cells. Embedded in a loop it finds and uses the largest detected cell for analysis by setting ROI to detected cell. The ROI is then drawn on a separate image and the area of the ROI is retrieved and stored. X and Y coordinates of ROI outline are stored as arrays and the centre of the cell is calculated. Angle and radius between centre of mass and outline are calculated.

Subsequently the average grey values for selected membranes along radius are stored and the pixels in the internal work image are deleted. Grey intensities for membranes in each channel are then normalized and the ratio between both channels is generated. Co-localization above threshold is measured and stored it in new array. Clusters (positive when adjacent channel co-localization positive) of co-localization are measured and the size and distribution along membrane calculated. The output consists of the generated results table with clusters the representation is drawn to new image.

Figure S4. Clustalw alignment of sequences of transmembrane regions from FGFR2 derived from four different species, and from three different FGFR-family members. * = Sequence identity; : = sequence similarity.

Figure S5. Original blots for Figures.

Supplementary Figure 1

Control blots

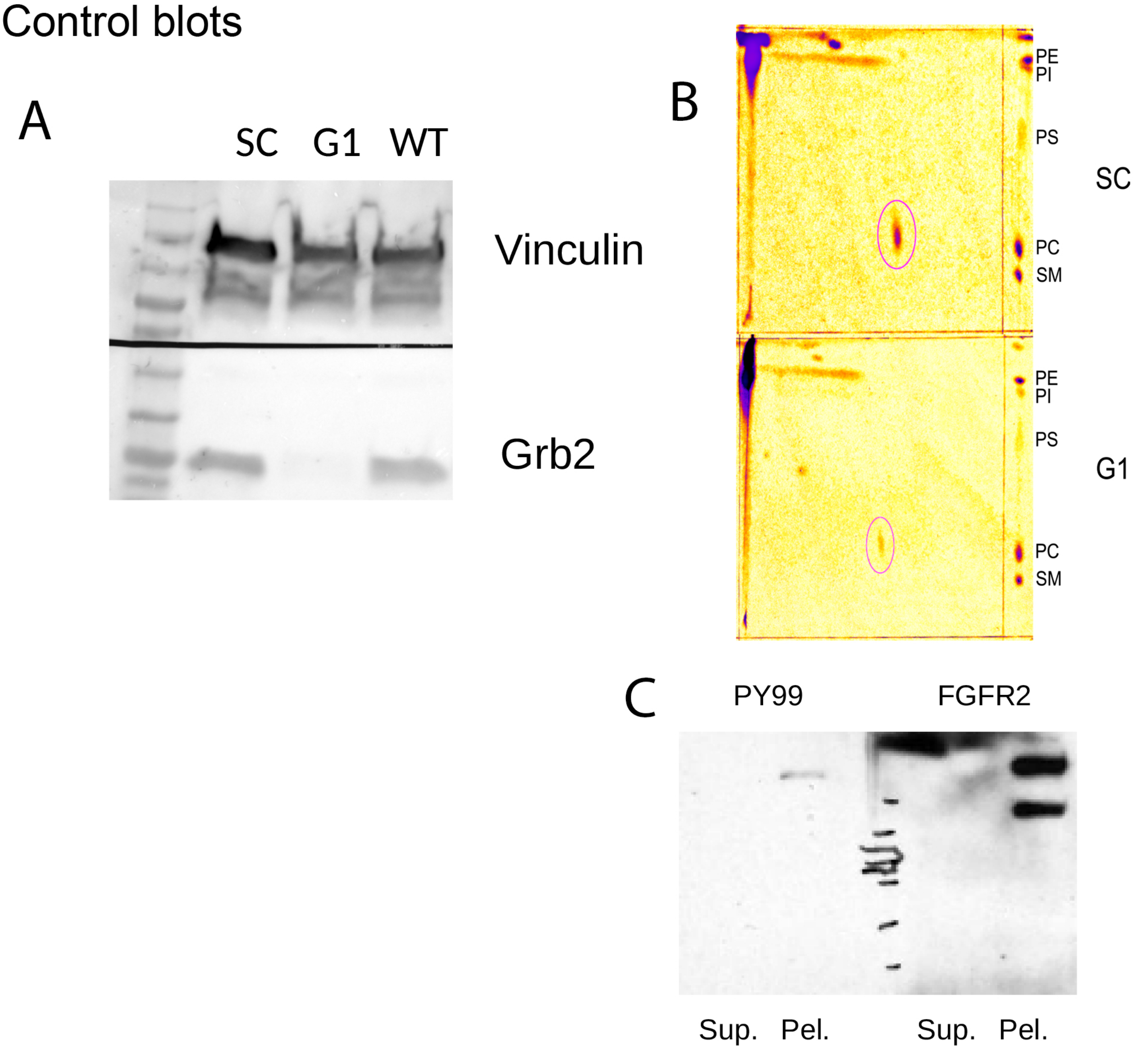

Supplementary Figure 2

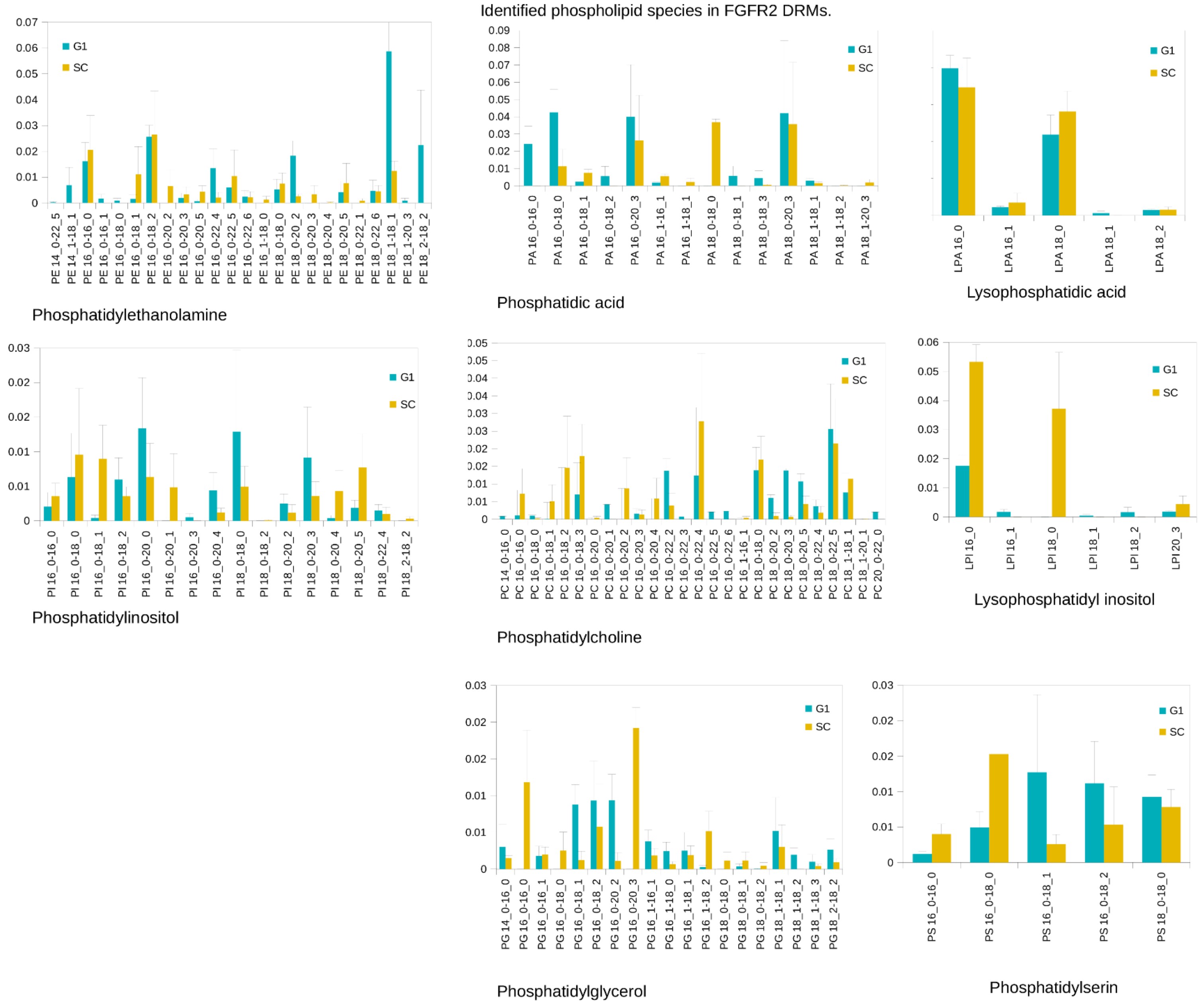

Supplementary Figure 3

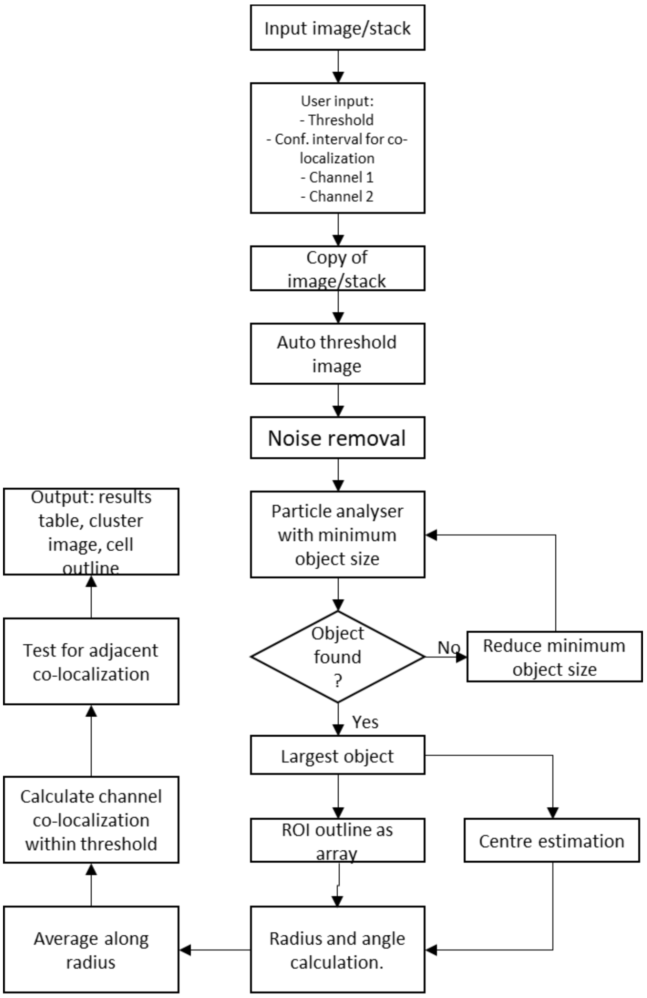

Workflow for Cluster 19 ImageJ plugin

Supplementary Figure 4

Clustalw alignment of FGFR2 transmembrane region

```
FGFR2_TM_Homo_sapiens                 IAIYCIGVFLIACMVVTVILC      Human
FGFR2_Erpetoichthys_calabaricus       IAIYCIGVFLIACMVVTVIVC      Fish
FGFR2_Falco_peregrinus                IAIYCIGVFLIACMVLTVILC      Bird
FGFR2_Chelonia_mydas                  IAIYCIGVFLIACMVLTVILC      Turtle
                                      ***************:***:*
```

```
FGFR2_Homo_sapiens       IAIYCIGVFLIACMVVTVILC
FGFR3_Homo_sapiens       ILSYGVGFFLFILVVAAVTLC
FGFR1_Homo_sapiens       IIIYCTGAFLISCMVGSVIVY
                         *  *  * **:  :* :* :
```

Supplementary Figure 5: Original Blots from Manuscript

Figure 4A. Sucrose gradient of membrane fractions blotted for FGFR2.

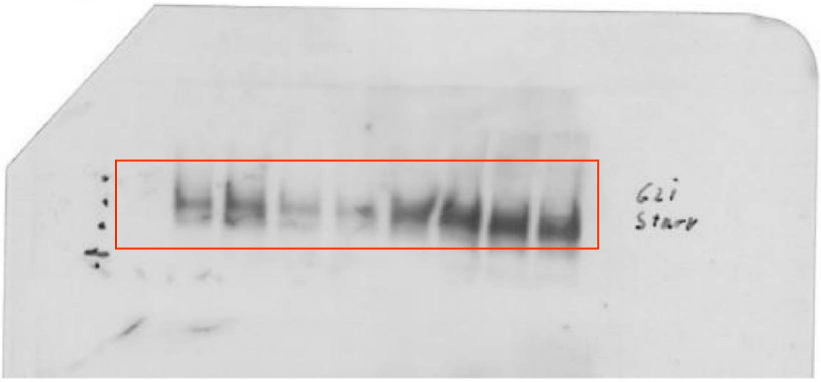

Figure 4A. Sucrose gradient of membrane fraction of FGFR2 in the presence of G1 starved and non-stimulated. Sucrose gradient increasing left to right. Blot inverted in Figure 4A. Red box includes bands used on second row Fig 4A.

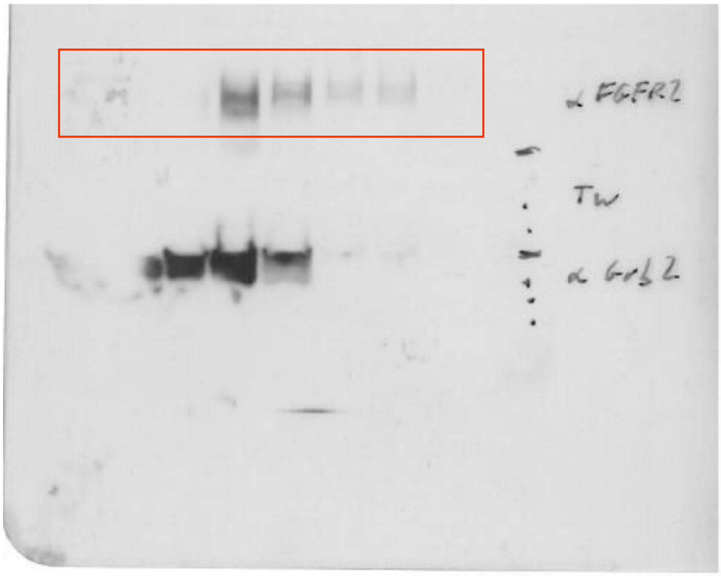

Figure 4A. Sucrose gradient of membrane fraction of FGFR2 in SC cells stimulated with FGF9. Sucrose gradient increasing left to right. Blot inverted in Figure 4A. Red box includes bands used on third row Fig 4A.

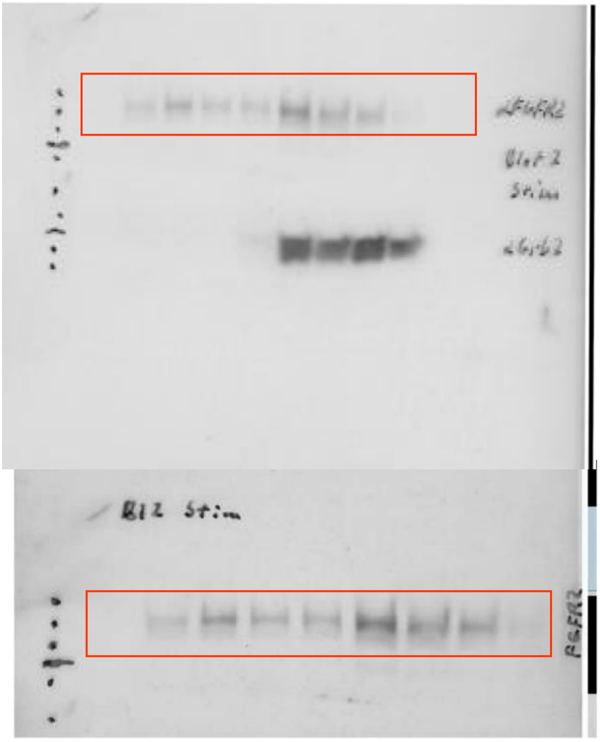

Figure 4A. Sucrose gradient of membrane fraction of FGFR2 in G1 cells stimulated with FGF9. Extended exposure below. Sucrose gradient increasing left to right. Blot inverted in Figure 4A. Red boxes include bands used on fourth row Fig 4A.

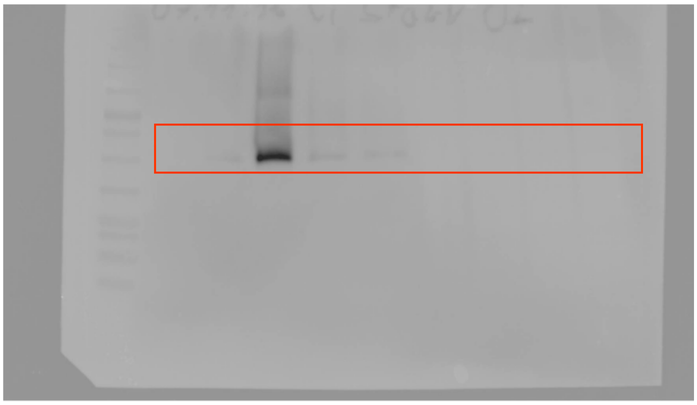

Figure 4A. Sucrose gradient of membrane fraction of FGFR2 blotted for Flotillin 1. Sucrose gradient increasing left to right. Blot inverted in Figure 4A. Red box includes bands used on fifth row Fig 4A.

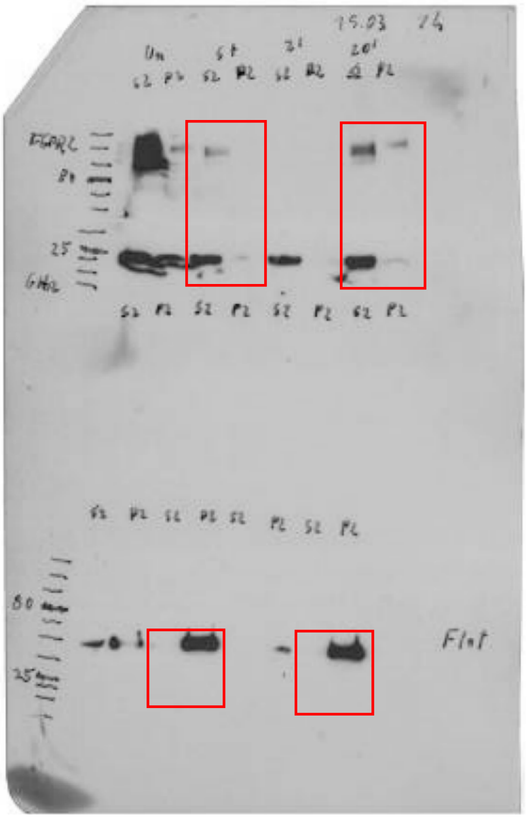

Figure 4B. Membrane fractions blotted for FGFR2, Grb2 (above) and Flotillin 1 (below) (lanes used in Figure marked in red). Blot marked with S-soluble fraction and P-membrane pellet fraction.

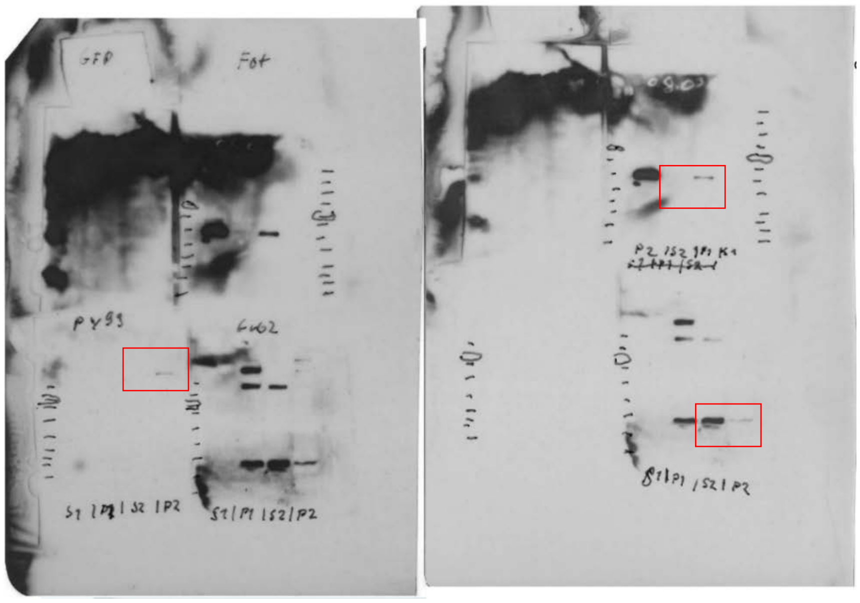

Figure 4C. Membrane fractions with from sucrose gradient. Blot on left – extended exposure. Membrane fraction blot for Flotillin 1 (above); py99 – showing phosphorylated FGFR2 (middle) and Grb2 (below). Lanes used in Figure marked in red. Blot marked with S-soluble fraction and P-membrane pellet fraction.

Figure 4D. Sucrose gradient of FGFR2 membrane fraction

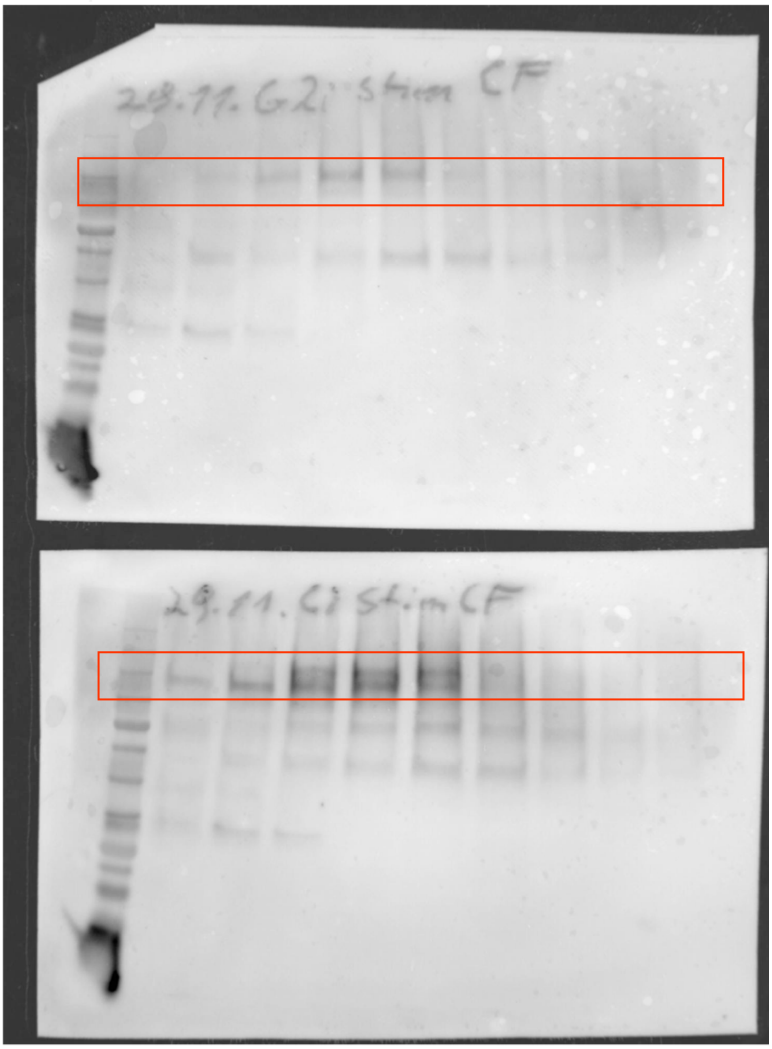

Figure 4D. Sucrose gradient of membrane fractions blotted for FGFR2. Top G1 cells stimulated with FGF9. Bottom SC cells stimulated with FGF9. Sucrose gradient increasing left to right. Red boxes include rows 2 and 1 respectively of Fig 4D

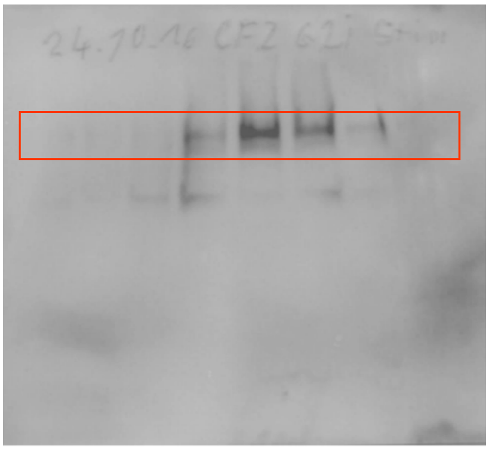

Figure 4D. Sucrose gradient of membrane fractions blotted for FGFR2. SC cells starved and non-stimulated. Sucrose gradient increasing left to right. Red box includes third row of Fig 4D.

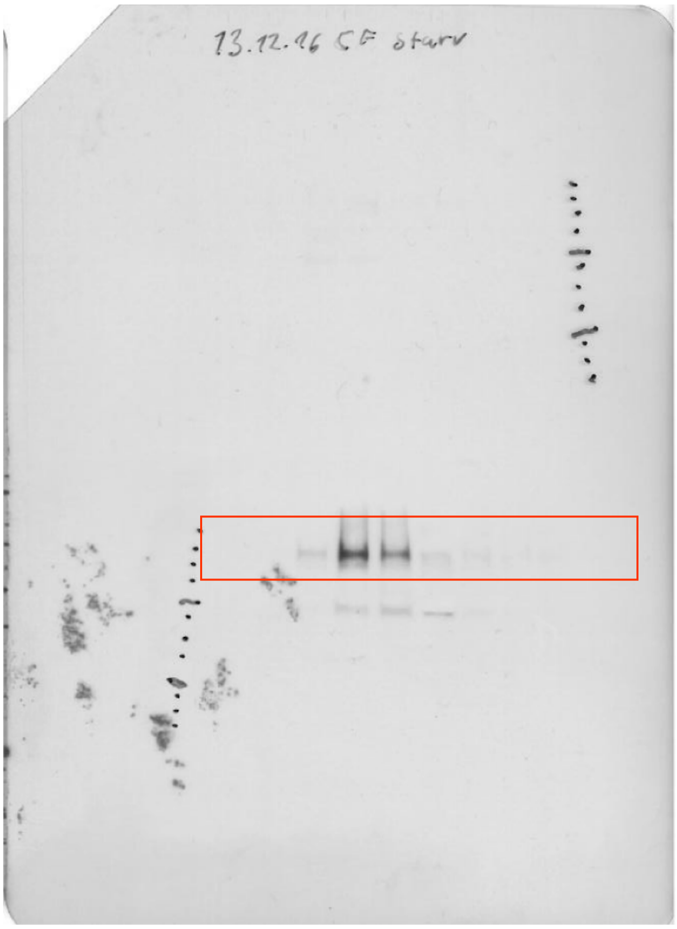

Figure 4D. Sucrose gradient of membrane fractions blotted for FGFR2. G1 cells starved and non-stimulated. Sucrose gradient increasing left to right. Red box includes fourth row of Fig 4D

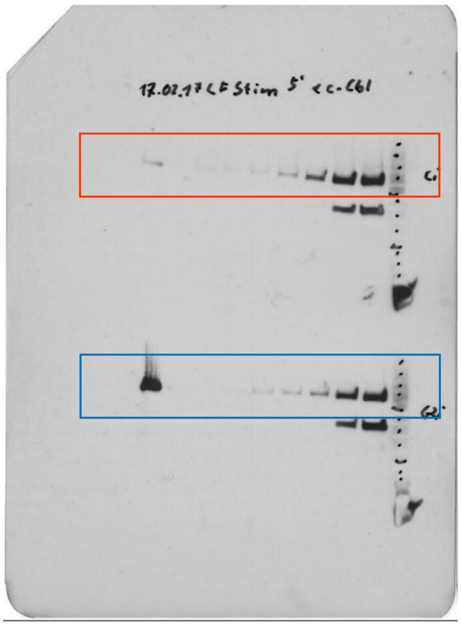

Figure 4D. Sucrose gradient of membrane fractions blotted for CBL. Top SC cells stimulated with FGF9 (Red Box). Bottom G1 cells stimulated with FGF9 (Blue Box). Sucrose gradient decreasing left to right.

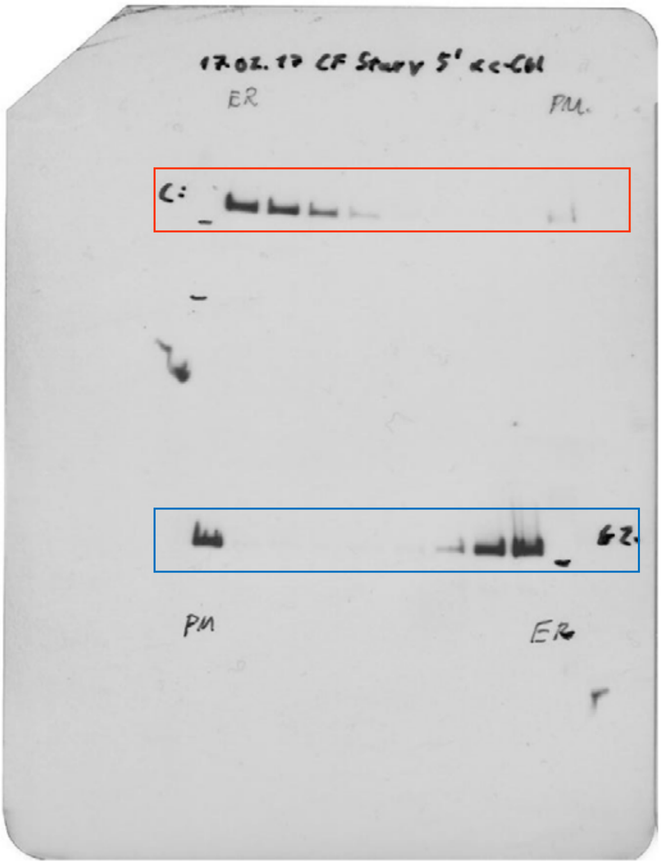

Figure 4D. Sucrose gradient of membrane fractions blotted for CBL. Top SC cells starved and non-stimulated (Red Box), sucrose gradient increasing left to right. Bottom G1 cells starved and non-stimulated (Blue Box), sucrose gradient decreasing left to right.

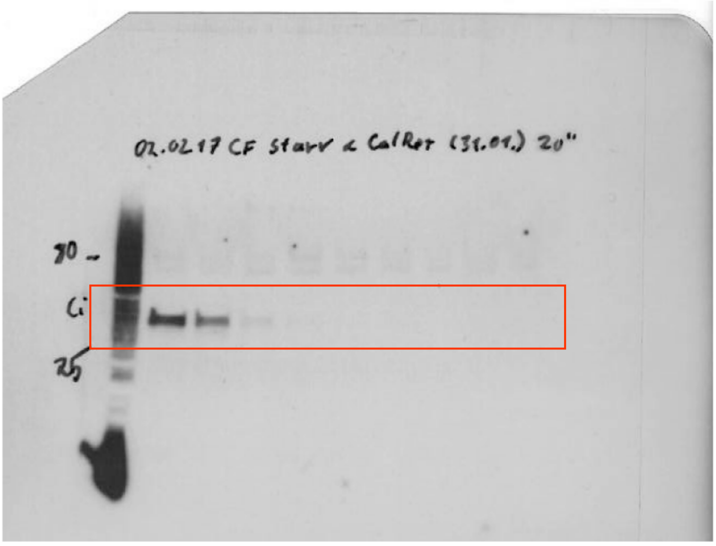

Figure 4D. Sucrose gradient of membrane fractions blotted for Calreticulin (Red Box). Sucrose gradient decreasing left to right.

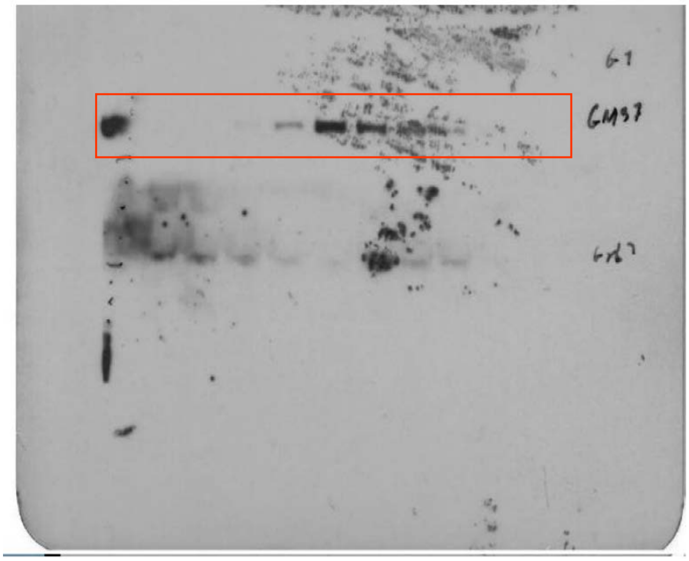

Figure 4D. Sucrose gradient of membrane fractions blotted for Golgin 97 (Red Box). Sucrose gradient increasing left to right.

Supplementary Material: Original Blots

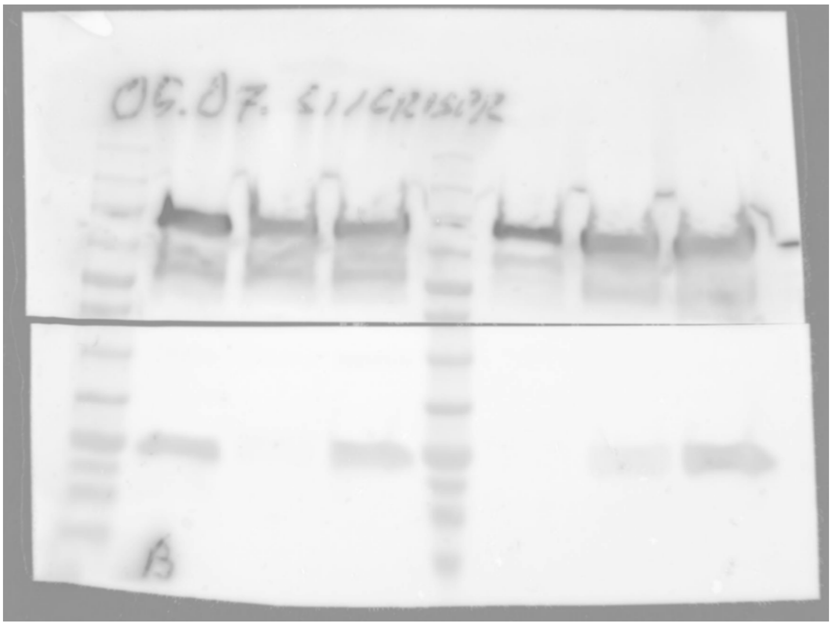

Supplementary Figure 1A Blot: Grb2 knock down.

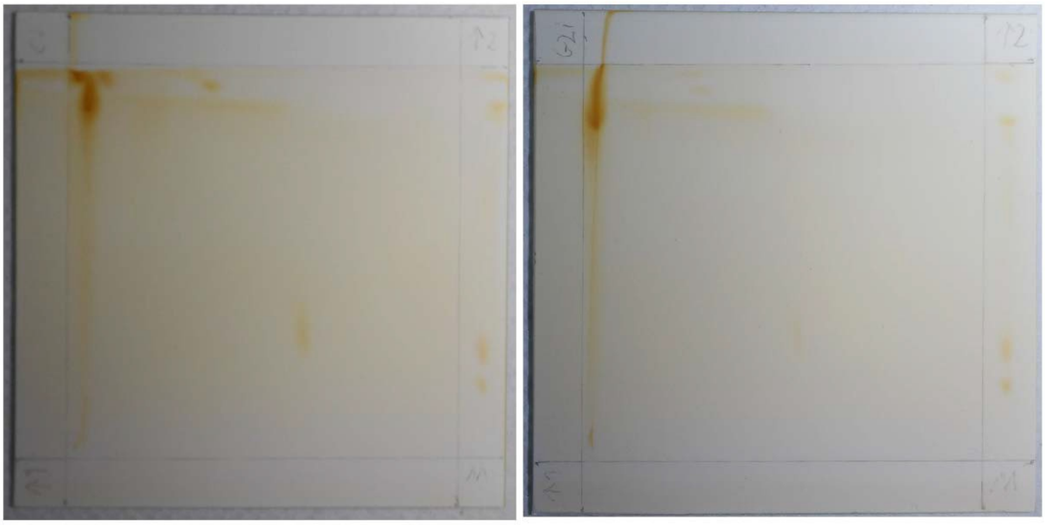

Supplementary Figure 1B. Independent quantification of PC from HEK293T cells using iodine vapour stained 2D HPTLC. Left: SC cells; Right G1 cells.

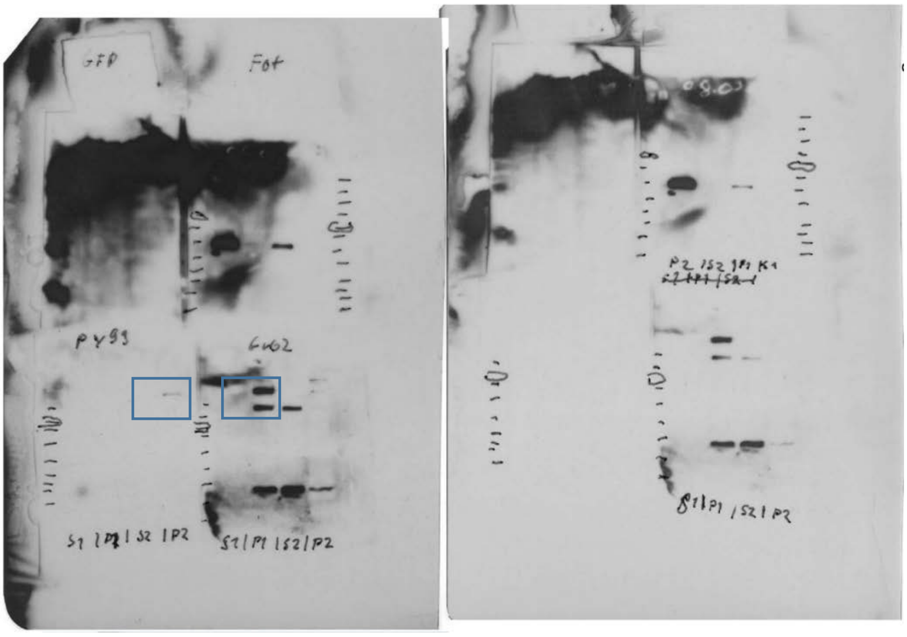

Supplementary Figure 1C. Membrane fractions from sucrose gradient. Blot on left – extended exposure. Membrane fraction blot anti-py99 (left - marked in blue) and anti-FGFR2 (right - marked in blue). Blot marked with S-soluble fraction and P-membrane pellet fraction.
